# Changes in ovarian reserve function after laparoscopic ovarian cystectomy: a retrospective cohort study

**DOI:** 10.3389/fsurg.2026.1822536

**Published:** 2026-06-22

**Authors:** Juan Yu, Sufen Liu, Chaoqun Wang, Ning Tao

**Affiliations:** Department of Gynecology, Aviation General Hospital, Beijing, China

**Keywords:** anti-Müllerian hormone, fertility outcome, follicle-stimulating hormone, laparoscope, ovarian cyst, ovarian reserve function

## Abstract

**Objective:**

To explore the effect of laparoscopic ovarian cystectomy on ovarian reserve function and its related risk factors.

**Methods:**

The clinical data of 150 patients with unilateral ovarian cysts admitted to our hospital were retrospectively analyzed. Paired t-tests were used to compare changes in indicators before and after surgery. One-way analysis of variance was performed to compare differences among different groups. Multivariable logistic regression analysis was used to identify independent risk factors for significant postoperative AMH decrease.

**Results:**

At 1, 3, 6, and 12 months after surgery, the AMH levels were significantly lower than those before surgery, while the FSH levels were higher than those before surgery (*P* < 0.05). At 12 months after surgery, the decrease in AMH levels and the increase in FSH levels in the cyst diameter ≥7 cm group were significantly greater than those in the cyst diameter <5 cm and 5 − 7 cm groups (*P* < 0.001). The decrease in AMH and the increase in FSH in the electrocoagulation hemostasis group were significantly greater than those in the suture hemostasis group (*P* < 0.05). Among 86 patients with fertility intentions, the overall pregnancy rate was 59.3% (51/86), and the live birth rate was 48.8% (42/86). Multivariable logistic regression analysis showed that cyst diameter ≥7 cm (OR = 3.610, 95%CI: 1.835 − 7.103), endometriotic cysts (OR = 2.063, 95%CI: 1.106–3.848), electrocoagulation hemostasis (OR = 2.633, 95%CI: 1.232 − 5.627), and moderate-to-severe ovarian tissue loss (OR = 4.697, 95%CI: 2.312 − 9.545) were independent risk factors for significant postoperative AMH decrease (≥30%) (*P* < 0.05).

**Conclusions:**

Laparoscopic ovarian cystectomy causes some damage to ovarian reserve function. After surgery, AMH levels decrease and FSH levels increase, but these levels gradually recover over time. Larger cyst diameter, endometriotic cysts, electrocoagulation hemostasis, and moderate-to-severe ovarian tissue loss are independent risk factors for postoperative ovarian reserve function damage.

## 1 Introduction

Ovarian cysts are one of the common gynecological diseases and may occur across all age groups, with a relatively high incidence among women of reproductive age. The detection rate of ovarian cysts in women of reproductive age is approximately 5%−7% and has shown a rising trend over the years with the widespread application of ultrasonography (1, 2). Ovarian cysts may cause symptoms such as abdominal pain, abdominal distension, and menstrual disorders, and may also lead to acute abdominal conditions such as cyst rupture and torsion, which severely affect patients’ quality of life and reproductive health (3). Laparoscopic ovarian cystectomy completely removes the cyst by dissecting the interface between the cyst wall and normal ovarian tissue. This approach can eliminate the lesion and maximize the preservation of normal ovarian tissue, and is particularly suitable for young patients with fertility intentions (4). However, the surgical procedures inevitably cause a certain degree of damage to the ovarian tissue, including mechanical damage to the ovarian cortex during dissection, impact of hemostatic manipulation on the ovarian blood supply, and follicular damage induced by inflammatory response. These factors may lead to a decline in ovarian reserve function after surgery (5).

Ovarian reserve refers to the quantity and quality of follicles remaining in the ovaries and reflects a woman's reproductive potential. The assessment of ovarian reserve function is particularly important for women of reproductive age and may be related to their fertility and reproductive health. Currently, commonly used clinical indicators for assessing ovarian reserve function include anti-Müllerian hormone (AMH) and follicle-stimulating hormone (FSH) (6). AMH is secreted by the granulosa cells of preantral and small antral follicles. AMH level is positively correlated with the follicular reserve in the ovary and is not affected by the menstrual cycle. It is currently recognized as the most sensitive and stable indicator for assessing ovarian reserve function. FSH is secreted by the anterior pituitary gland and regulates follicular development through a negative feedback mechanism. When ovarian reserve declines, FSH levels increase (7, 8). The combined detection of AMH and FSH can more comprehensively and accurately assess ovarian reserve function. Recent studies have shown that AMH levels decrease to varying degrees following ovarian cystectomy, while FSH levels increase correspondingly, suggesting that ovarian reserve function may be impaired after the procedure. However, there are still only a few studies on what factors affect the degree of change in these indicators.

Based on the above background, this retrospective cohort study systematically analyzed the dynamic changes in ovarian reserve indicators (AMH and FSH) at different time points after laparoscopic ovarian cystectomy. It investigated the impact of cyst size, cyst type, hemostasis method, and degree of ovarian tissue loss on postoperative ovarian reserve, and analyzed the relationship between postoperative changes in ovarian reserve and fertility outcomes. The aim was to provide a scientific basis for optimizing surgical strategies, reducing postoperative ovarian reserve impairment, and improving fertility prognosis.

## 2 Material and methods

### 2.1 Study design

This is a retrospective cohort study. The research protocol was approved by the ethics committee of Aviation General Hospital. The study complied with the requirements of the Declaration of Helsinki. All patients and their families signed the informed consent form. The clinical data of patients who underwent laparoscopic unilateral ovarian cystectomy in the gynecology department of our hospital between August 2023 and October 2024 were retrospectively collected through the hospital information system.

Inclusion criteria: (1) women of reproductive age (18–45 years old); (2) diagnosis of unilateral ovarian cysts through preoperative ultrasound or MRI examination, with cyst diameter ≥3 cm; (3) Undergoing laparoscopic ovarian cystectomy, with pathological diagnosis confirmed as benign ovarian cysts after surgery; (4) completion of at least one AMH and FSH measurement before and after surgery; (5) complete medical records, including preoperative baseline data, surgical records, and postoperative follow-up data.

Exclusion criteria: (1) patients with bilateral ovarian cysts; (2) history of ovarian surgery; (3) postoperative pathological diagnosis of malignant or borderline tumors; (4) presence of endocrine disorders such as polycystic ovary syndrome, premature ovarian failure, and ovarian hypofunction; (5) use of medications affecting ovarian function within 3 months before surgery (e.g., gonadotropin-releasing hormone agonists, oral contraceptives); (6) severe cardiac, hepatic, or renal dysfunction or other systemic diseases; (7) severe pelvic adhesions during surgery, requiring combined surgery; (8) loss to follow-up or incomplete data.

According to the above screening criteria, a total of 150 patients were finally included in the study.

### 2.2 Surgical methods

All surgeries were performed by three gynecologic surgeons with extensive experience in laparoscopic procedures, which reduced operative heterogeneity and potential bias, thus enhancing the reliability of the surgical data. All surgeons underwent standardized preoperative training and strictly adhered to the following standardized surgical procedures: (1) Patients were placed in the lithotomy position. Following general anesthesia, routine disinfection and draping were performed. (2) Pneumoperitoneum was established at the umbilicus, and the intra-abdominal pressure was maintained at 12 − 15 mmHg. A 10 mm trocar and laparoscope were inserted to explore the pelvic cavity. (3) A 5-mm trocar and a 10-mm trocar were inserted into the lower abdomen on both sides as operating ports. (4) The affected ovary was fully exposed, and the cyst was fixed with atraumatic forceps. A 2–3 cm incision was made in the ovarian cortex at the site of greatest surface tension of the cyst. (5) Blunt and sharp dissections were performed along the interface between the cyst wall and normal ovarian tissue to completely remove the cyst. The cyst was then placed in a specimen bag and removed. (6) The ovarian wound was explored, and the residual cyst wall tissue was removed. (7) The hemostasis method was selected based on the degree of bleeding: electrocoagulation hemostasis (using bipolar electrocoagulation, power 25 − 30 W, punctate electrocoagulation), suture hemostasis (using absorbable sutures to intermittently or continuously suture the ovarian wound), or electrocoagulation combined with suture hemostasis. (8) The pelvic cavity was thoroughly irrigated, and no active bleeding was confirmed before closure of the incisions. The operation time, blood loss, hemostasis method, and degree of normal ovarian tissue loss were recorded in detail during the procedure.

Evaluation criteria for the degree of normal ovarian tissue loss: Based on intraoperative findings and postoperative pathological examination, the loss of normal ovarian tissue was classified into mild (<10%, smooth cyst removal, with minimal loss of normal ovarian tissue), moderate (10%−30%, with a small amount of normal ovarian tissue removed along with the cyst wall during removal), and severe (>30%, with the cyst tightly adhered to the ovarian tissue, difficult removal, and significant loss of normal ovarian tissue) (9).

### 2.3 Outcome measures

#### 2.3.1 Primary outcome measures

(1) Surgery-related indicators were collected, including operation time, intraoperative blood loss, hemostasis method (electrocoagulation hemostasis, suture hemostasis, electrocoagulation combined with suture hemostasis), degree of normal ovarian tissue loss (mild, moderate, severe), intraoperative complications, conversion to open surgery, and postoperative length of stay. (2) Ovarian reserve function indicators: AMH and FSH levels were measured before surgery and at 1, 3, 6, and 12 months after surgery. All patients underwent fasting blood collection in the morning on days 2 − 5 of their menstrual cycle (early follicular phase). Serum AMH levels were measured using chemiluminescence immunoassay (reagent kit: Beckman Coulter, normal reference range: 2.0 − 6.8 ng/mL). Serum FSH levels were measured using electrochemiluminescence immunoassay (reagent kit: Roche Diagnostics, normal reference range: 3.0 − 8.0 mIU/mL). The change values and change rates of AMH and FSH levels were calculated at various time points after surgery.

#### 2.3.2 Secondary outcome measures

(1) AMH decline value and rate: AMH decline value = preoperative AMH−postoperative AMH. A significant decline in AMH was defined as a decline rate of ≥30% at 12 months after surgery. (2) Fertility outcome: Patients with fertility intentions after surgery were followed up. The methods of conception attempted (natural conception or assisted reproductive technology), pregnancy status, early miscarriage, ectopic pregnancy, live birth, and time to pregnancy were recorded. The total pregnancy rate = number of pregnancies/total number of patients with fertility intentions×100%. The live birth rate = number of live births/total number of patients with fertility intentions×100%.

### 2.4 Follow-up measures

All patients were followed up through regular outpatient visits or telephone follow-up at 1, 3, 6, and 12 months after surgery. During each follow-up visit, menstrual status, fertility intention, pregnancy status, and serum AMH and FSH levels were recorded. For patients lost to follow-up, contact attempts were made by telephone, WeChat, or other means to obtain relevant information.

### 2.5 Statistical methods

Statistical analyses were performed using SPSS 26.0 statistical software. Continuous data conformed to a normal distribution and were expressed as mean ± standard deviation. Paired samples t-tests were used for preoperative and postoperative comparisons. One-way analysis of variance (ANOVA) was used for comparisons among multiple groups. Least significant difference (LSD)-t tests were used for pairwise comparisons between groups. Categorical data were expressed as number of cases and percentages [n (%)], and *χ*^2^ tests or Fisher's exact test were used for comparisons between groups. Multivariable logistic regression analysis was used to identify independent risk factors for significant postoperative AMH decrease, with an AMH decline rate ≥30% at 12 months postoperatively as the dependent variable. *P* < 0.05 was considered statistically significant.

## 3 Results

### 3.1 Baseline data of patients

A total of 150 patients with unilateral ovarian cysts who met the inclusion criteria were included in this study. The baseline characteristics are shown in Table 1.

**Table 1:** Baseline characteristics of patients (mean ± SD)/ [n (%)].

Baseline characteristics	Overall (*n* = 150)
Age (years)	32.68 ± 5.70
BMI (kg/m^2^)	22.75 ± 3.11
Gravidity	1.06 ± 0.75
Parity	0.53 ± 0.55
Nulligravida (cases)	37
Cyst laterality (left/right) (cases)	73/77
Cyst diameter (cm)	5.62 ± 1.69
Cyst types	
Mature teratoma (cases)	62 (41.33)
Endometriotic cyst (cases)	48 (32.00)
Serous cystadenoma (cases)	21 (14.00)
Mucinous cystadenoma (cases)	14 (9.33)
Others (cases)	5 (3.33)
Preoperative AMH (ng/mL)	3.30 ± 1.42
Preoperative FSH (mIU/mL)	6.82 ± 2.15

### 3.2 Surgery-related indicators of included patients

The surgery-related indicators of all patients were statistically analyzed, and the results are shown in Table 2.

**Table 2:** Surgery-related indicators of patients (mean ± SD)/ [n (%)].

Surgery indicator	Result (*n* = 150)
Operation time (min)	78.80 ± 24.34
Intraoperative blood loss (mL)	75.93 ± 33.18
Hemostasis methods	
Electrocoagulation hemostasis (cases)	92 (61.33)
Suture hemostasis (cases)	35 (23.33)
Electrocoagulation + suture (cases)	23 (15.33)
Degree of normal ovarian tissue loss	
Mild (<10%) (cases)	67 (44.67)
Moderate (10%–30%) (cases)	58 (38.67)
Severe (>30%) (cases)	25 (16.67)
Intraoperative complications (cases)	8 (5.33)
Conversion to open surgery (cases)	2 (1.33)
Postoperative length of stay (d)	3.23 ± 1.15

### 3.3 Changes in AMH levels before surgery and at various time points after surgery

**Figure 1:**
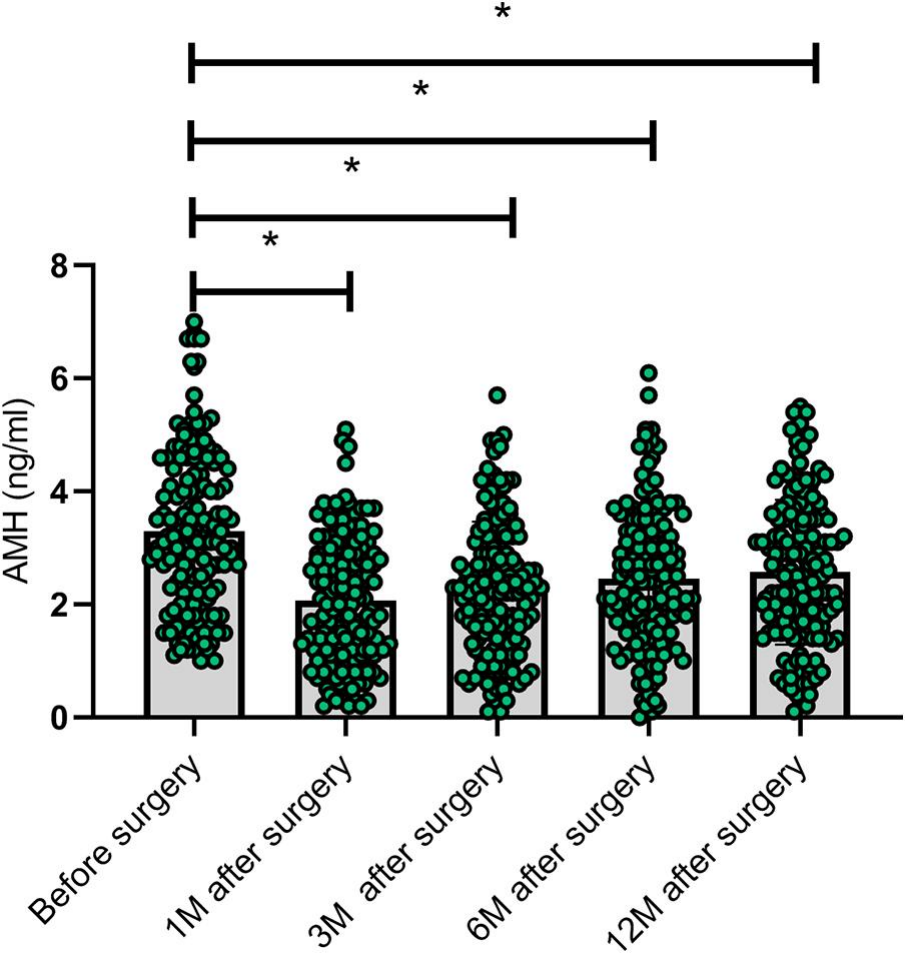
Changes in AMH levels before surgery and at various time points after surgery. At 1, 3, 6, and 12 months after surgery, the AMH levels of patients were significantly lower than those before surgery (*P* < 0.05). Asterisk (*) indicates statistically significant difference between groups.

The AMH levels at 1, 3, 6, and 12 months after surgery were significantly reduced than those before surgery (*P* < 0.05), as shown in Table 3 and Figure 1.

**Table 3:** Changes in AMH levels before surgery and at various time points after surgery (mean ± SD).

Time point	AMH level (ng/mL)	t	P	Change rate compared with before surgery (%)
Before surgery	3.30 ± 1.42	-		
1 month after surgery	2.06 ± 1.11	8.426	<0.001	−24.04 ± 56.42
3 months after surgery	2.28 ± 1.18	6.766	<0.001	−14.78 ± 67.36
6 months after surgery	2.46 ± 1.23	5.476	<0.001	−7.80 ± 66.23
12 months after surgery	2.57 ± 1.28	4.677	<0.001	−3.84 ± 72.04

Paired samples t-tests were used for comparison with those before surgery.

### 3.4 Changes in FSH levels before surgery and at various time points after surgery

**Figure 2:**
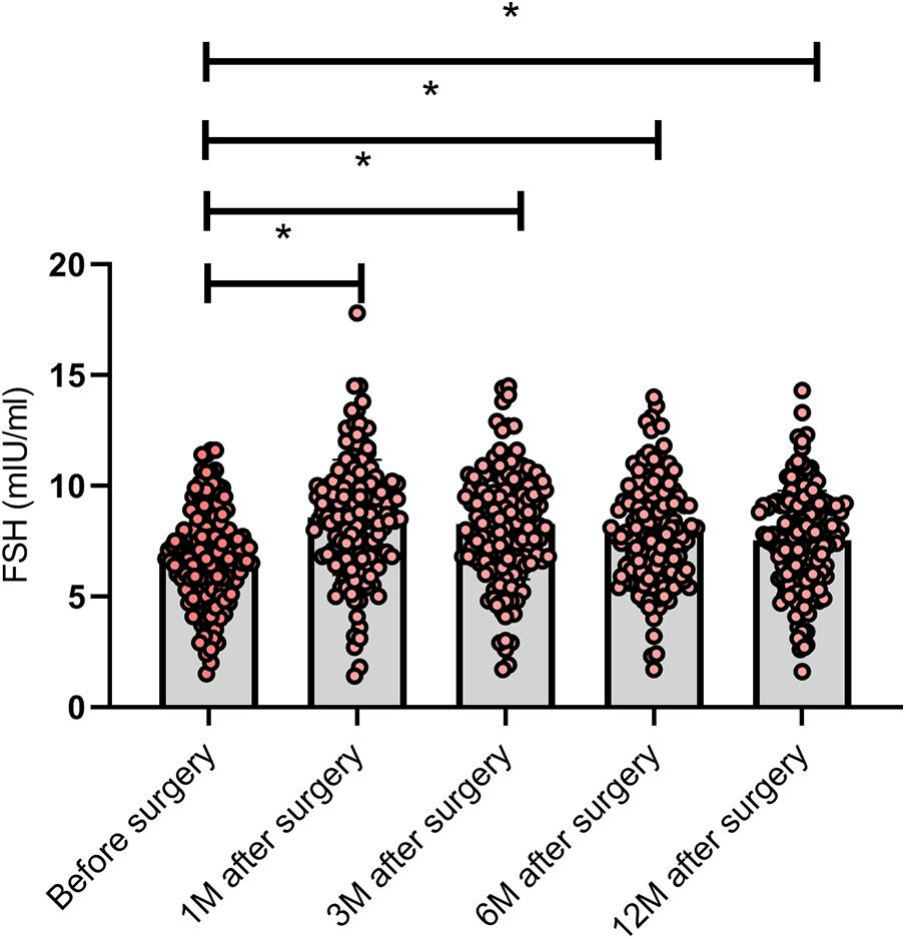
Changes in FSH levels before surgery and at various time points after surgery. At 1, 3, 6, and 12 months after surgery, the FSH levels of patients were significantly higher than those before surgery (*P* < 0.05). Asterisk (*) indicates statistically significant difference between groups.

The FSH levels at 1, 3, 6, and 12 months after surgery were significantly elevated than those before surgery (*P* < 0.05), as shown in Table 4 and Figure 2.

**Table 4:** Changes in FSH levels before surgery and at various time points after surgery (mean ± SD).

Time point	FSH level (mIU/mL)	t	P	Change rate compared with before surgery (%)
Before surgery	6.82 ± 2.15	-		
1 month after surgery	8.58 ± 2.60	6.389	<0.001	45.37 ± 94.15
3 months after surgery	8.26 ± 2.49	5.361	<0.001	35.85 ± 62.90
6 months after surgery	7.93 ± 2.35	4.268	<0.001	36.06 ± 86.98
12 months after surgery	7.52 ± 2.26	2.748	0.006	24.97 ± 68.12

Paired samples t-tests were used for comparison with those before surgery.

### 3.5 Comparison of postoperative ovarian reserve function changes in patients with different cyst diameters

**Figure 3:**
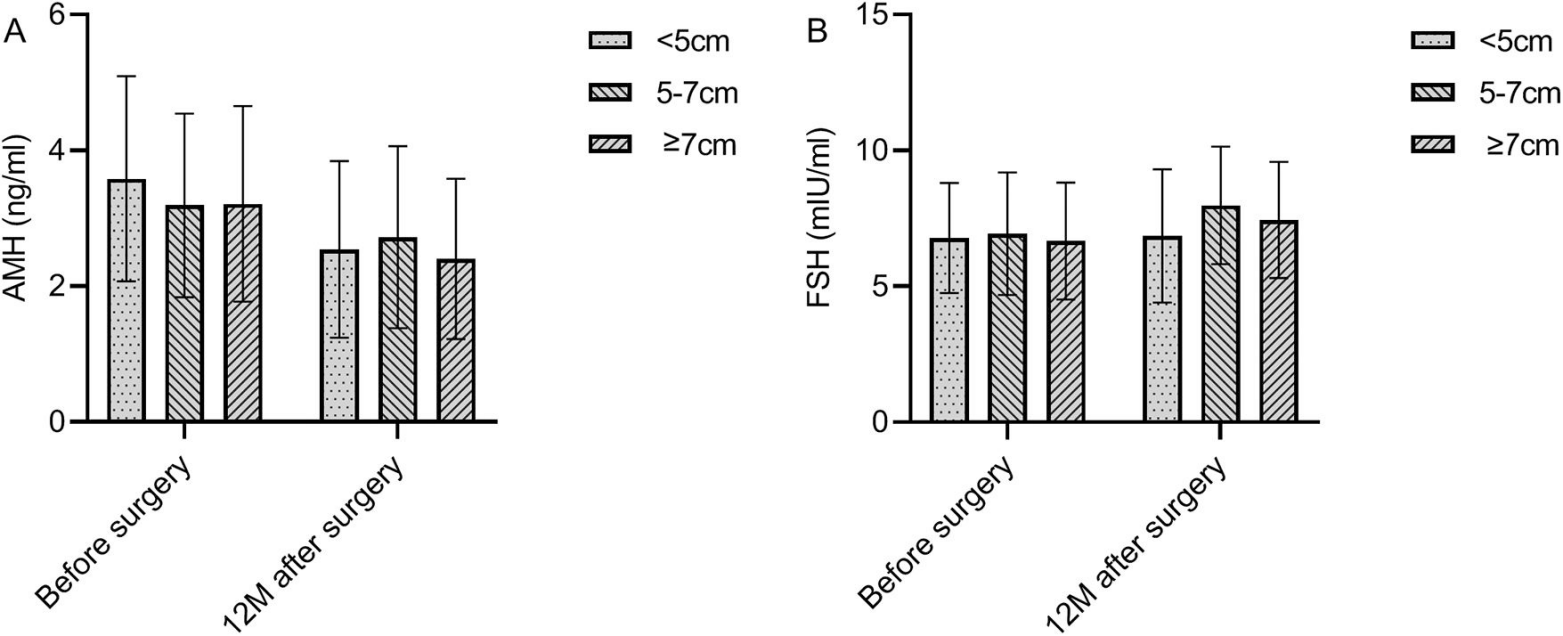
Comparison of postoperative ovarian reserve function changes in patients with different cyst diameters. At 12 months after surgery, the decrease in AMH levels **(A)** and the increase in FSH levels **(B)** in the cyst diameter ≥7 cm group were significantly greater than those in the cyst diameter <5 cm and 5–7 com groups (*P* < 0.05), indicating that larger cyst diameters were associated with more pronounced ovarian reserve impairment. AMH: anti-Müllerian hormone; FSH: follicle-stimulating hormone.

Patients were classified into <5 cm, 5 − 7 cm, and ≥7 cm groups according to cyst diameter. The changes in AMH and FSH levels at 12 months after surgery were compared among the three groups. The results showed that larger cyst diameters were associated with more pronounced ovarian reserve impairment after surgery (*P* < 0.05), as shown in Table 5 and Figure 3.

**Table 5:** Comparison of postoperative ovarian reserve function changes in patients with different cyst diameters.

Indicator	<5 cm (*n* = 42)	5 − 7 cm (*n* = 65)	≥7 cm (*n* = 43)	F	P
AMH before surgery (ng/mL)	3.58 ± 1.51	3.19 ± 1.35	3.21 ± 1.44	0.584	0.559
AMH at 12 months after surgery (ng/mL)	2.54 ± 1.30	2.72 ± 1.34	2.40 ± 1.18	5.247	0.006
AMH decline value (ng/mL)	0.47 ± 2.00	0.80 ± 1.76	1.23 ± 2.03	19.745	<0.001
FSH before surgery (mIU/mL)	6.78 ± 2.02	6.94 ± 2.26	6.67 ± 2.15	0.147	0.863
FSH at 12 months after surgery (mIU/mL)	6.86 ± 2.45	7.98 ± 2.16	7.44 ± 2.14	2.347	0.099
FSH elevation value (mIU/mL)	0.08 ± 3.46	0.77 ± 3.01	1.04 ± 2.77	5.684	0.004

### 3.6 Comparison of postoperative ovarian reserve function changes in patients with different hemostasis methods

**Figure 4:**
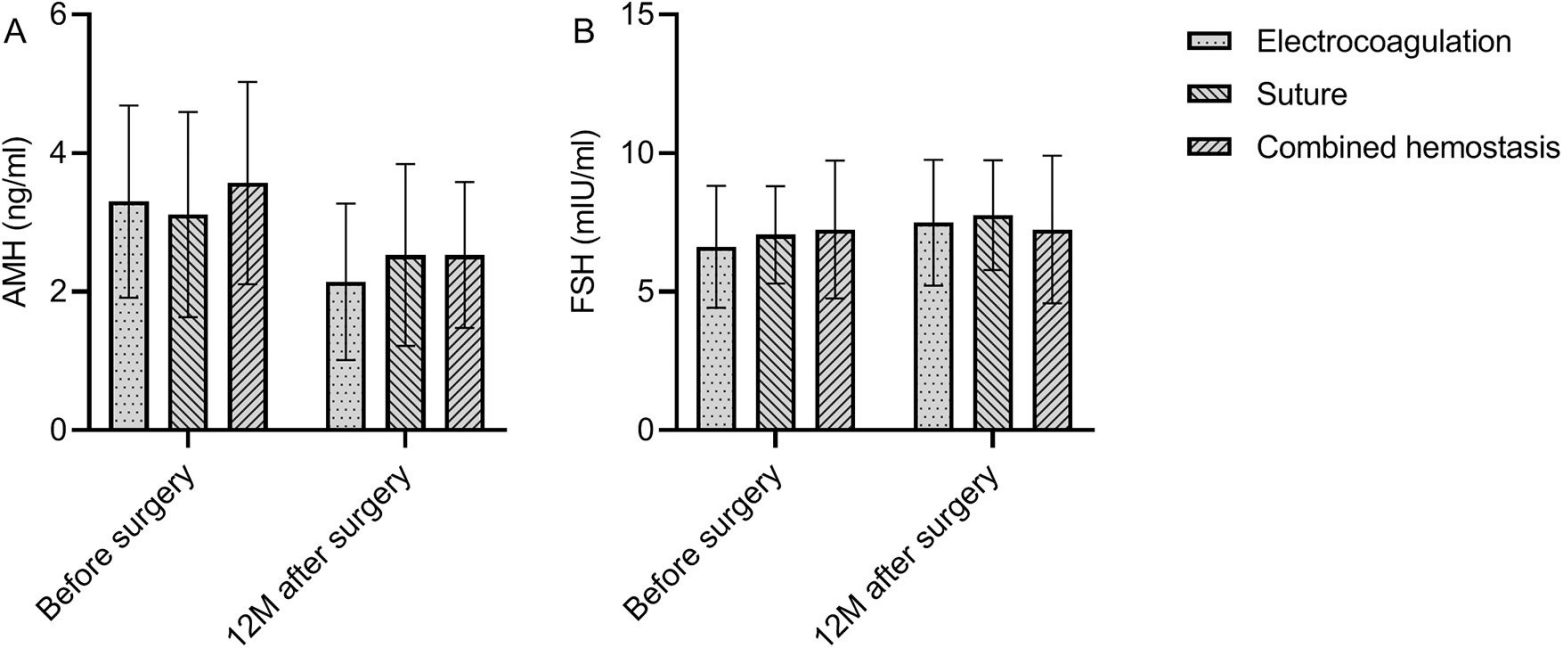
Comparison of postoperative ovarian reserve function changes in patients with different hemostasis methods. At 12 months after surgery, the decrease in AMH (**A**) and the increase in FSH (**B**) in the electrocoagulation hemostasis group were significantly greater than those in the suture hemostasis group (*P* < 0.05). AMH: anti-Müllerian hormone; FSH: follicle-stimulating hormone.

Patients were classified into an electrocoagulation hemostasis group, a suture hemostasis group, and an electrocoagulation + suture group according to the intraoperative hemostasis methods. The changes in ovarian reserve function at 12 months after surgery were compared among the three groups. The results showed that the decrease in AMH and the increase in FSH in the electrocoagulation hemostasis group were significantly greater than those in the suture hemostasis group (*P* < 0.05), as shown in Table 6 and Figure 4.

**Table 6:** Comparison of postoperative ovarian reserve function changes in patients with different hemostasis methods.

Indicator	Electrocoagulation (*n* = 92)	Suture (*n* = 35)	Electrocoagulation + Suture (*n* = 23)	F	P
AMH before surgery (ng/mL)	3.30 ± 1.39	3.11 ± 1.48	3.57 ± 1.46	0.247	0.781
AMH at 12 months after surgery (ng/mL)	2.14 ± 1.13	2.53 ± 1.31	2.53 ± 1.05	2.184	0.116
AMH decline value (ng/mL)	1.04 ± 1.90	0.57 ± 1.81	0.71 ± 1.86	8.947	<0.001
FSH before surgery (mIU/mL)	6.62 ± 2.20	7.05 ± 1.75	7.24 ± 2.49	0.124	0.883
FSH at 12 months after surgery (mIU/mL)	7.49 ± 2.27	7.76 ± 1.98	7.24 ± 2.66	1.647	0.195
FSH elevation value (mIU/mL)	0.87 ± 1.18	0.35 ± 1.21	0.62 ± 1.07	3.247	0.042

### 3.7 Analysis of postoperative fertility outcomes

Patients with fertility intentions after surgery (*n* = 86) were followed up for 18.42 ± 6.58 months. The postoperative pregnancy status and live births were statistically analyzed, and the results are shown in Table 7.

**Table 7:** Analysis of postoperative fertility outcomes [n (%)].

Indicator	Result (*n* = 150)
Patients with fertility intention	86 (100.00)
Postoperative attempts at natural conception (cases)	71 (82.56)
Successful natural conception (cases)	42 (59.15)
Failed natural conception (cases)	29 (40.85)
Postoperative assisted reproductive technology (cases)	15 (17.44)
Successful ART conception (cases)	9 (60.00)
Failed ART conception (cases)	6 (40.00)
Overall pregnancy rate	51 (59.30)
Early miscarriage (cases)	7 (13.73)
Ectopic pregnancy (cases)	2 (3.92)
Live birth (cases)	42 (82.35)
Live birth rate	42 (48.84)
Time to pregnancy (months)	8.67 ± 5.24

### 3.8 Multivariable analysis of factors influencing AMH decline rate after surgery

With the AMH decline rate of ≥30% at 12 months after surgery as the dependent variable, factors with *P* < 0.10 in the univariable analysis were included in the multivariable logistic regression analysis. The results showed that cyst diameter ≥7 cm, electrocoagulation hemostasis, and moderate-to-severe ovarian tissue loss were independent risk factors for significant postoperative AMH decrease (*P* < 0.05), as shown in Table 8.

**Table 8:** Multivariable analysis of factors influencing AMH decline rate after surgery.

Risk factor	B	S.E	Wald	OR	95% CI	P
Age ≥35 years	0.547	0.328	2.784	1.728	0.909–3.286	0.095
BMI ≥28 kg/m^2^	0.386	0.412	0.879	1.471	0.656–3.298	0.348
Cyst diameter ≥7 cm	1.284	0.345	13.847	3.610	1.835–7.103	<0.001
Endometriotic cyst	0.724	0.318	5.185	2.063	1.106–3.848	0.023
Electrocoagulation hemostasis	0.968	0.387	6.254	2.633	1.232–5.627	0.012
Moderate-to-severe ovarian tissue loss	1.547	0.362	18.274	4.697	2.312–9.545	<0.001
Operation time ≥90 min	0.612	0.341	3.222	1.844	0.945–3.599	0.073
Intraoperative blood loss ≥100 mL	0.438	0.356	1.514	1.550	0.771–3.115	0.219

## 4 Discussion

### 4.1 Impact of laparoscopic ovarian cystectomy on ovarian reserve function

This study systematically analyzed the dynamic changes in ovarian reserve function at different time points after surgery in 150 patients with unilateral ovarian cysts. The results showed that AMH levels at 1, 3, 6, and 12 months after surgery were significantly lower than those before surgery, while FSH levels were correspondingly higher. This suggests that laparoscopic ovarian cystectomy causes some damage to ovarian reserve function, which is consistent with previous studies. Further analysis revealed that the AMH levels of patients in this study initially decreased and then increased after surgery, reaching their lowest point at 1 month after surgery and then gradually increasing. However, they still could not return to preoperative levels at 12 months after surgery. This dynamic change suggests that ovarian cystectomy has the most significant impact on the ovarian reserve function of patients in the early stages. The residual ovarian tissue may partially restore ovarian reserve function due to compensatory hyperplasia over time.

In this study, mature teratomas (dermoid cysts) accounted for 41.33% of all cyst types, with a relatively high proportion. This phenomenon may be related to the following factors. First, mature teratomas are one of the most common benign ovarian tumors in in women of reproductive age, and they account for a relatively high proportion among patients in this age group in China. Second, the inclusion criteria for this study were women of reproductive age with cysts ≥3 cm in diameter. Mature teratomas, due to the distinct imaging features (fatty echoes and hyperechoic nodules visible on ultrasound), have a relatively high proportion of cases detected and recommended for surgery. In addition, as this study was conducted at a single tertiary care center, the composition of referred patients may differ from that of the general community population, potentially leading to an overrepresentation of specific cyst types. This is an inherent selection bias of single-center studies, and it is recommended that this be taken into account when generalizing the findings of this study to other populations.

The decline in ovarian reserve function after surgery is attributed to several factors. First, the surgical operation directly damages the ovary, especially during cyst wall dissection. The unclear boundary between the cyst and normal tissue may lead to inadvertent excision, thereby damaging the ovarian cortex. The ovarian cortex is one of the main sources of AMH. Cortical damage may directly lead to reduction in the number of follicles and decrease in AMH level (10, 11). Second, intraoperative hemostatic manipulation may affect ovarian blood supply, thereby leading to decline in ovarian reserve function. For instance, electrocoagulation hemostasis in this study may affect the ovarian artery or the ovarian branches of the uterine artery, causing local ischemia and hypoxia, leading to follicular apoptosis or ovarian atrophy (12). Third, the local inflammatory response caused by surgical trauma also affects the ovarian reserve function. The intense inflammatory response can damage the granulosa cells, thereby inhibiting the secretion of AMH (13). In this study, the amplitude of changes in FSH was more stable compared with that in AMH, possibly because FSH is regulated by more factors.

In patients with endometriosis, the impact of surgery on fertility is of particular concern. Ovarian endometriomas are tightly adherent to ovarian tissue, and surgical excision may lead to unavoidable damage to the normal ovarian cortex. In addition, the chronic inflammatory state of endometriosis may exert a persistent detrimental effect on ovarian function. Previous research has shown that ovarian reserve is already reduced before surgery in patients with endometriosis and may decline further after surgery (14). These findings suggest that individualized surgical strategies should be developed after carefully balancing the potential benefits of surgery against the risk of ovarian damage. Furthermore, the use of energy-based devices during laparoscopic surgery may have a significant impact on ovarian reserve. Thermal damage caused by bipolar electrocoagulation during surgery may affect the blood vessels in hilum of ovary and adjacent ovarian cortex, resulting in follicular loss. Evidence suggests that during ovarian cystectomy, appropriate control of coagulation power, reduction of activation time and coagulation area, or the use of suturing instead of electrocoagulation for hemostasis may help minimize ovarian tissue damage and preserve residual ovarian reserve (15). The results of this study are consistent with the above evidence and further support the importance of optimizing the use of energy-based devices in clinical practice.

### 4.2 Impact of cyst diameter on postoperative ovarian reserve function

In this study, patients were classified into three groups based on cyst diameters: <5 cm, 5 − 7 cm, and ≥7 cm. The results showed that larger cyst diameters were associated with greater decline value in AMH and elevation value in FSH at 12 months after surgery. Multivariable logistic regression analysis further confirmed that cyst diameter ≥7 cm was an independent risk factor for significant postoperative AMH decrease (OR = 3.610). This finding suggests that large cysts have more significant impact on postoperative ovarian reserve function. Cyst diameter may affect postoperative ovarian reserve function through several mechanisms. First, larger cysts exert greater compression on surrounding normal ovarian tissue. Prolonged compression may lead to thinning of the ovarian cortex and reduced follicular density, thereby affecting ovarian function (16). Second, larger cysts bring more difficulties during dissection and cause greater damage to normal ovarian tissue. Third, hemostasis range may increase after the removal of large cysts, which is consistent with the aforementioned point that hemostatic manipulation may aggravate insufficient blood supply to the ovary. Fourth, removal of larger cysts may require longer surgical duration, prolonging ovarian exposure to the pneumoperitoneum environment. Prolonged carbon dioxide exposure may cause oxidative stress damage to the ovarian tissue (17, 18).

Based on the above findings, we suggest that for ovarian cysts with large diameters, clinicians should fully weigh the pros and cons. Early surgical intervention, preoperative assessment of the dissection surface, precise intraoperative surgical techniques, and optimization of hemostasis methods may help reduce damage to normal ovarian tissue and maximize preservation of residual ovarian reserve function.

### 4.3 Impact of hemostasis method on postoperative ovarian reserve function

This study compared the effects of different hemostasis methods on postoperative ovarian reserve function. The results showed that the decrease in AMH in the electrocoagulation hemostasis group at 12 months after surgery was significantly greater than that in the suture hemostasis group. Multivariable analysis confirmed that electrocoagulation hemostasis was an independent risk factor for significant postoperative AMH decrease (OR = 2.633). The findings indicate that the choice of hemostasis methods has a significant impact on postoperative ovarian reserve function. Electrocoagulation hemostasis causes more damage to ovarian reserve function than suture hemostasis. Electrocoagulation hemostasis has the advantages of simplicity and rapid effectiveness, and is the most commonly used hemostasis method in laparoscopic surgery (19). However, the high temperature generated during electrocoagulation not only coagulates local tissue proteins to achieve hemostasis, but also causes thermal damage to surrounding tissues. The heat generated by electrocoagulation may also spread to the blood vessels in hilum of ovary, leading to vasospasm, endometrial damage, or even thrombosis, thereby affecting ovarian blood supply (20, 21). Reduced ovarian blood flow can lead to ischemia and hypoxia in follicles and cause follicular atresia and apoptosis, ultimately leading to decreased AMH levels (22). In contrast, suture hemostasis achieves hemostasis by mechanically closing blood vessels in the wound without causing thermal damage, thereby causing relatively less trauma to the ovarian tissue. Suture hemostasis also contributes to the recovery of ovarian morphology and the healing of the wound. In this study, the decrease in AMH in the suture hemostasis group was significantly lower than that in the electrocoagulation hemostasis group. This verifies the advantage of suture hemostasis in protecting ovarian reserve function. However, suture hemostasis also has some limitations, including high technical requirements for manipulation, relatively long operation time, and relatively slow onset of hemostasis compared with electrocoagulation. The results of this study showed that the operation time of the suture hemostasis group was indeed significantly longer than that of the electrocoagulation hemostasis group. This is a factor that needs to be weighed in clinical practice.

The decline value in AMH in the electrocoagulation combined with suture hemostasis group was between that in the electrocoagulation and suture hemostasis groups, suggesting that the combined use of the two hemostasis methods may be a compromise. For areas with significant bleeding, electrocoagulation can be used initially for hemostasis, followed by suturing to further close the wound. This combined method both ensures hemostatic effect and reduces thermal damage from electrocoagulation to some extent. Based on the findings of this study, it is recommended that the use of electrocoagulation be minimized during the procedure, especially in the hilum of the ovary and the cortical region rich in follicles. Electrocoagulation should be performed in a punctate and intermittent manner to avoid large-area, prolonged electrocoagulation operations. The power should be set at a low level, and bipolar electrocoagulation should be used rather than monopolar electrocoagulation to reduce the heat diffusion range (23, 24). Of course, the choice of hemostasis method should also be comprehensively considered based on the patient's specific condition, cyst characteristics, intraoperative bleeding condition, and the surgeon's technical expertise.

### 4.4 Impact of degree of ovarian tissue loss and cyst type on ovarian reserve function

In this study, the risk factor analysis revealed that moderate-to-severe ovarian tissue loss was an independent risk factor for significant postoperative AMH decrease, and it has the highest odds ratio (OR) among all risk factors. This result suggests that protecting normal ovarian tissue is important for maintaining postoperative ovarian reserve. To minimize the loss of ovarian tissue, the following points are recommended: First, preoperative ultrasound or MRI should be used to thoroughly assess the nature and size of the cyst. Second, during the procedure, it is advisable to choose an appropriate anatomical layer for dissection. For cysts with unclear boundaries, dissection may start from the relatively well-defined areas, and the range is gradually expanded. Third, excessive traction or compression of ovarian tissue should be avoided during dissection. Regarding the impact of cyst type on ovarian reserve, the results of this study suggest that endometriotic cysts are an independent risk factor for postoperative AMH reduction, which is similar to previous research results. Endometriosis is a chronic inflammatory condition in which ectopic endometrial tissue bleeds repeatedly in the ovary, forming old cysts that compress normal ovarian tissue. Therefore, the ovarian reserve function of patients may have been damaged to some extent before surgery (25, 26). Meanwhile, the endometriotic cyst walls are often tightly adhered to normal ovarian tissue, lacking clear anatomical layers, making surgical removal difficult. This suggests that a more cautious surgical strategy should be adopted for patients with endometriotic cysts. For younger patients with fertility intensions, if the cyst is small, drug intervention can be considered. When surgery is necessary, meticulous operation should be performed, accompanied by proper follow-up after surgery.

The significant changes in ovarian reserve markers observed in this study indicate that preoperative fertility preservation should be an integral part of multidisciplinary treatment decisions for high-risk patients, such as those with cysts ≥7 cm in diameter or endometriotic cysts. Oocyte or embryo cryopreservation is a proven method of fertility preservation, particularly suitable for patients whose ovarian reserve is already impaired prior to surgery or who are at high risk of ovarian damage. Guidelines from the European Society of Human Reproduction and Embryology and the American Society for Reproductive Medicine recommend that patients for whom surgery may significantly affect ovarian reserve should receive preoperative reproductive counseling and be fully informed about fertility preservation options (27, 28). Therefore, for high-risk patients, it is recommended that preoperative consultation with a reproductive endocrinologist be incorporated into standard clinical practice to fully assess ovarian reserve and discuss individualized fertility preservation strategies, particularly among young patients who have not yet completed their fertility planning.

In this study, the levels of AMH and FSH exhibited dynamic changes postoperatively, with the most pronounced recovery occurring within 3–6 months after surgery. This finding has important clinical implications for guiding the timing of assisted reproductive treatment in patients who did not undergo fertility preservation prior to surgery. For patients who have experienced significant impairment of ovarian reserve following surgery and still require assisted reproductive technology to conceive, under the conditions where age and condition permit, it may be advisable to initiate the assisted reproductive technology cycle 3–6 months after surgery, once ovarian reserve has partially recovered, in order to achieve better ovarian response and oocyte quality. However, this strategy must be weighed against the risk of recurrence, particularly for patients with endometriotic cysts, as the risk of postoperative recurrence increases over time, and waiting too long may further damage ovarian reserve. Therefore, for patients of advanced reproductive age or those with endometriosis, whose ovarian reserve is already impaired, it is recommended that they be referred to a reproductive medicine department as soon as possible after surgery to avoid missing the optimal window for assisted reproduction while waiting for recovery. In general, the timing of postoperative assisted reproductive intervention should be individually assessed based on patient age, baseline ovarian reserve, cyst type, risk of recurrence, and patient preference, seeking an optimal balance between the potential benefits of waiting for partial recovery of ovarian reserve and the risk of recurrence associated with delayed treatment.

It should also be noted that patient age is an important confounding factor affecting postoperative AMH levels. The mean age of the study population was 32.68 ± 5.70 years. In the multivariable logistic regression analysis, age ≥ 35 years was included as a covariate. The results showed that age ≥ 35 years was associated with an OR of 1.728 [95% confidence interval (CI): 0.909–3.286; *P* = 0.095], which did not reach statistical significance. These findings suggest that in this study population, age may have a limited independent effect on a significant postoperative decline in AMH levels, which may be related to the sample size and age distribution. However, since AMH levels naturally decline with age, patients of advanced reproductive age tend to have lower preoperative baseline AMH levels, and the clinical significance of postoperative decline is more pronounced. Therefore, individualized assessment and thorough preoperative counseling should be conducted for patients of advanced reproductive age in clinical practice.

### 4.5 Analysis of postoperative fertility outcomes

The follow-up results showed that the overall pregnancy rate of the included patients was 59.30%. This finding suggests that although laparoscopic ovarian cystectomy causes some damage to ovarian reserve, most patients still achieve successful pregnancy and delivery after surgery. It indicates that the impact of the surgery on fertility outcomes is limited.

### 4.6 Research clinical implication and surgical optimization strategies

Based on the findings of this study, the following surgical optimization strategies are proposed for patients requiring laparoscopic ovarian cystectomy, especially younger patients with fertility intensions. First, it is necessary to conduct a thorough preoperative assessment, evaluate patient's reproductive history and fertility requirements in detail, use multiple methods to assess cysts, develop an individualized surgical plan, and inform patients of the surgical risks. Second, surgical indications should be strictly evaluated. For asymptomatic patients with small cysts and normal ovarian reserve, close follow-up is recommended. For those who require surgery, medication may be considered prior to surgery. Third, meticulous intraoperative procedures are performed, making full use of microsurgical techniques to properly remove the cyst and minimize damage to the normal ovary. Fourth, hemostatic measures should be optimized to minimize electrocoagulation manipulation. When necessary, punctate and intermittent electrocoagulation may be combined with suture hemostasis. Finally, close postoperative follow-up should be conducted. If necessary, fertility guidance can be provided, and intervention from a reproductive medicine center can be sought.

### 4.7 Research limitations and prospects

This study has some limitations. First, this study was a single-center retrospective analysis with a limited sample size, which may have led to selection bias and restricted extrapolation of the results. Second, the follow-up period was relatively short, and the long-term trend of changes in ovarian reserve function after surgery still needs further analysis. Third, no quantitative analysis was conducted on the technical details such as the specific electrocoagulation time, power, and suture method during surgery, and no more refined surgical recommendations were proposed. Finally, postoperative fertility outcomes were not comprehensively assessed. To address these shortcomings, it is planned to conduct multi-center, large-sample prospective cohort studies or follow-up controlled trials and extend the follow-up period to 2 − 3 years after surgery or longer. This allows more comprehensive assessment of changes in postoperative ovarian reserve function. Additionally, more clinical indicators can be considered for multi-dimensional assessment of ovarian reserve function.

Laparoscopic ovarian cystectomy causes some damage to ovarian reserve function. After surgery, AMH levels decrease and FSH levels increase. The impairment was most pronounced at 1 month after surgery and gradually recovered over time, but it still did not fully recover to preoperative levels at 12 months postoperatively. Cyst diameter ≥7 cm, endometriotic cysts, electrocoagulation hemostasis, and moderate-to-severe ovarian tissue loss are independent risk factors for postoperative ovarian reserve function decline.

## Data Availability

The original contributions presented in the study are included in the article/Supplementary Material, further inquiries can be directed to the corresponding author.
